# Quantification of Flavonoids, Phenols and Antioxidant Potential from Dropped *Citrus reticulata* Blanco Fruits Influenced by Drying Techniques

**DOI:** 10.3390/molecules26144159

**Published:** 2021-07-08

**Authors:** Dinesh Kumar, Milind S. Ladaniya, Manju Gurjar, Sunil Kumar, Sachin Mendke

**Affiliations:** ICAR-Central Citrus Research Institute, Nagpur 440 033, Maharashtra, India; msladaniya@gmail.com (M.S.L.); manjugurjar28@yahoo.com (M.G.); sunil_ut2004@yahoo.co.in (S.K.); sachinmendke@ymail.com (S.M.)

**Keywords:** *Citrus reticulata* Blanco, immature dropped fruits, drying techniques, flavonoids, phenols, antioxidants, nutraceutical

## Abstract

Physiologically dropped immature *Citrus reticulata* Blanco fruits are regarded as waste and discarded in the citrus orchard but are a good source of bioactive compounds including flavonoids, antioxidants and total phenols. A study was undertaken to identify and quantify these bioactive compounds and to investigate the influence of different drying techniques, namely freeze drying and hot air oven drying, on flavonoids namely flavanone glycosides, antioxidant potential and total phenol content in immature dropped fruits of *Citrus reticulata* Blanco. Flavonoids were quantified in high-performance liquid chromatography (HPLC). The antioxidant activity were investigated with three assays azino-*bis* [3-ethylbenzthiazoline-6-sulfonic acid]) (ABTS), 2,2-diphenyl-1-picrylhydrazyl radical (DPPH), Ferric Reducing Ability of Plasma (FRAP) and total phenol content was determined. Freeze dried samples of 12 and 14 mm size retained maximum hesperidin flavonoid content (27.03% and 27.20%) as compared to the hot air dried samples (17.99%) and retained higher phenolic content ranged from 50.54–54.19 mg GAEL^−1^. The antioxidant activity in freeze dried fruits was from 12.21–13.55 mM L^−1^ Trolox and 15.27–16.72 mM L^−1^ Trolox with ABTS, DPPH assay and FRAP values ranging from 7.31–9.07 mM L^−1^ Trolox. Significant positive correlation was found between the flavonoid hesperidin with antioxidant assays and total phenolic content (TPC). The results showed that waste citrus fruits can act as potential source of bioflavonoids, especially hesperidin, and antioxidants for pharmaceutical as well as nutraceutical industry.

## 1. Introduction

Citrus fruits are among the most cultivated horticultural fruit crop. They belong to the *Rutaceae* family and encompass fruits of varying sizes and shapes. Major citrus groups include mandarins (*C. reticulata*), sweet oranges (*C. sinensis*), sour/bitter oranges (*C. aurantium*), lemons (*C. limon*), limes (*C. aurantifolia*), grapefruit (*C. paradisi*), citrons (*C. medica*), pummelos (*C. grandis*) and many hybrids [1]. Citrus fruits are cultivated in around 140 countries and India ranks fourth in world citrus fruit production. The overall production is about 13.20 million tonnes [2]. The top five citrus producing countries are China, Brazil, USA, India, Mexico and Spain. Citrus fruits have found many applications in food, cosmetics, beverages and pharmaceutical industries [3]. Health benefits of citrus are well known. Therapeutic properties of citrus fruits include anti-viral, anti-cancer, anti-inflammatory, anti-oxidant, etc. These benefits are associated with range of phytochemicals and bioactive compounds like flavonoids, vitamins, carotenoids, phenols, minerals, etc.that are present [4].

Physiological dropping of immature fruits is a natural phenomenon and have two dropping times. The first dropping occurs due to degeneracy, imperfect pollination, ovule dysplasia or malnutrition. The second dropping is characterized by the changes of endogenous hormones caused due to early embryo ripening. Physiologically dropped immature fruits are the small, young immature fruits and green in color abscised due to physiological reasons from either the stem–branch or ovary–stem junctions [5]. The considerable amounts of phytochemical compounds (flavonoids, limonoids, and synephrine) and antioxidant compounds are found in immature fruits in comparison to mature fruits [5,6,7].

Flavonoids can be classified into three major groups: flavanones, flavones and flavonols. Citrus flavanones are either present in the glycosides form or aglycone forms. Naringenin and hesperitin are in the aglycone forms. Glycoside forms are of two types: neohesperidosides and rutinosides. Naringin, neohesperidin and neoeriocitrin comes under neohesperidosides and have a bitter taste. Rutinosides include hesperidin, narirutin and didymin which are tasteless. The typical taste of citrus fruits is due to the presence of flavanones in diglycosides form [8]. Accumulation of flavonoid content in citrus species occurs during the developmental stages. However the presence and distribution is highly variable and depends on a number of genetic and environmental factors which affect the concentration of bioactive components present in different species and varieties [9]. Hesperidin, the flavanone glycoside, is the characteristic flavonoid found in the genus *citrus*. It consists of hesperitin and sucrose (glucose and rhamnose) named as rutinose. The highest amount of hesperidin is usually found in young tissues [10]. Citrus fruits exhibit potent antioxidant activity due to presence of range of phytochemicals like phenolic compounds, flavonoids, carotenoids, vitamins, etc. [3,11]. The antioxidant compounds scavenge or block free radicals like singlet oxygen (^1^O_2_), hydroxyl radical (OH·), superoxide anion (O_2_), peroxy radical (R–O–O·), etc. responsible for causing oxidative stress, inhibits oxidation of various biomolecules [4] and which reduce the risk of cardiovascular diseases, coronary heart diseases, arteriosclerosis and even some forms of cancer [12]. Phenolic compounds present in citrus species scavenge reactive oxygen radicals; have anti-bacterial as well as anti-inflammatory properties and also interrupts the chain reaction in the lipid peroxidation process. They also affect the aroma and color of food. Phenols display health promoting effect by modifying the metabolic activities of humans [13,14]. The immature citrus fruits are usually discarded and dumped as waste but currently of great interest and have found applications in pharmaceutical and food industries due to its tremendous nutritional value [15]. The bioactive compounds of fruits and vegetables having nutraceutical properties are delivered orally by encapsulating the bioactive agents in suitable delivery system [16]. The microemulsions, molecular complexes, liposomes, emulsions, microgels, nanoparticles, biopolymer particles are popularly used colloidal delivery systems. The pharmaceutical industry uses solubilising technology namely the use of nanoparticles, mucolytics and intestinal permeation enhancers (PE) for weakly soluble nutraceuticals and for effective delivery [17].

These unwanted fruits rich in bioactive compounds are a rich source of value added food supplements and provide a platform for producing nutraceuticals which are inexpensive, efficient and eco-friendly [11]. Dropped citrus fruits are usually sold in dry form. A traditionally employed drying method include sun drying is relatively simple and inexpensive but not feasible all year round. However, these techniques can lead to oxidation of some nutritional components present. Oven drying is affordable on the industrial scale and the temperature conditions can be monitored easily. The technique is also available during off-season. The freeze drying technique has advantage in preserving the nutritional value as well as sensory quality but can lead to loss of some active ingredients. The drying techniques have different effects on bioactive and phytochemical compounds and vary with the species involved [5,18]. To the best of our knowledge, very scarce information is available regarding the utilization of dropped immature mandarin fruits and the influence of different drying methods on phytochemical content and antioxidant potential.

A study was thus undertaken to see the influence of drying methods on flavonoids and phenolic content as well as antioxidant potential of mandarin (*Citrus reticulata* Blanco) of different size of dropped immature fruits in order to evaluate comparative efficiency of the techniques and obtain the maximum yield. The information obtained will be useful in extraction of bioactive compounds having nutraceutical importance.

## 2. Results and Discussion

**Figure 1:**
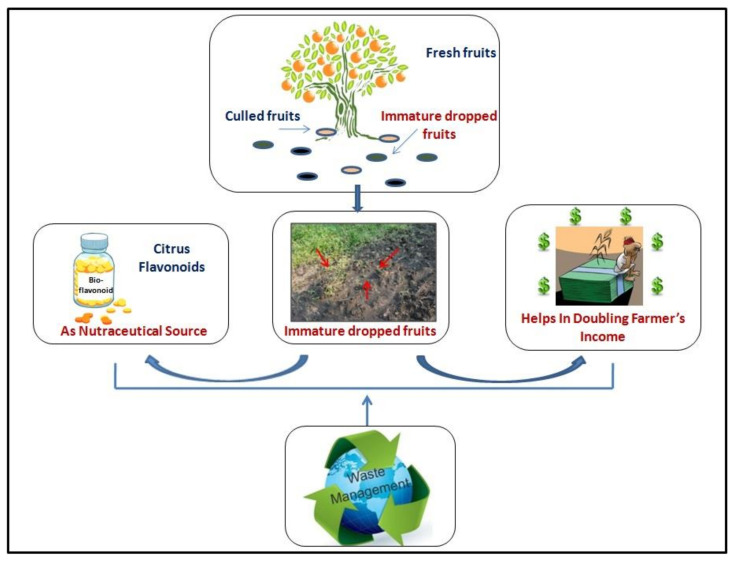
Pictorial abstract of the study.

Figure 1 is the graphical abstract summarizing the study findings.

### 2.1. Flavonoids Content

**Figure 2:**
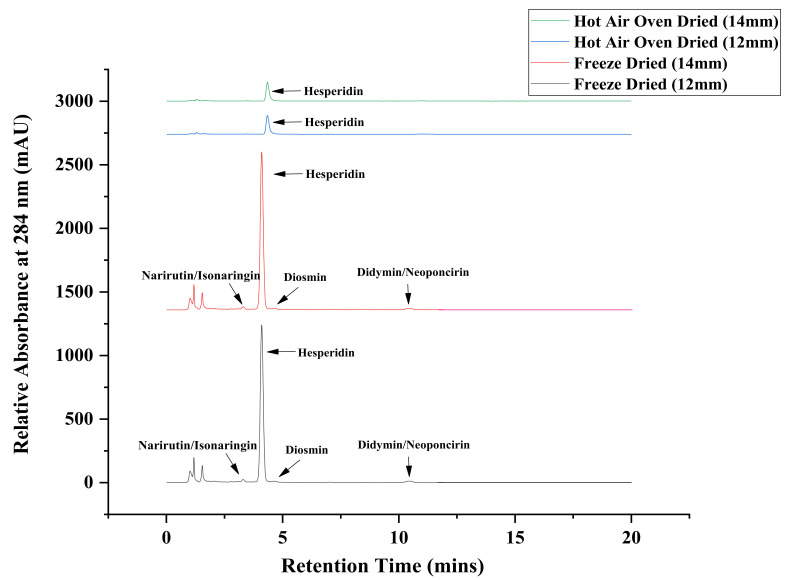
High-performance liquid chromatography (HPLC) chromatogram of flavonoids content of 12 mm and 14 mm physiologically dropped immature fruits after freeze drying and hot air oven drying.

The major flavonoids found in dropped immature *Citrus reticulata* Blanco fruits was hesperidin. Flavonoids belong to the benzopyrene derivatives class and exhibit potent antioxidant activity. They are ubiquitously present in plants [19]. The flavonoids content (relative percentage) determined are reported in Table 1. Among flavonoids determined, hesperidin is one of the major flavanone glycosides. Hesperidin content varies with the citrus species [20]. The hesperidin content ranged on dry weight basis from 8.08–28.48% after freeze drying and 5.27–17.99% after hot air drying technique in varying sizes from 8–24 mm of immature dropped mandarin fruits. Different drying techniques had significant effects on the flavonoid content at *p* < 0.01. It can be seen from the table that the highest content of hesperidin (20.93–28.48% in freeze drying and from 10.94–17.99% in hot air drying) was obtained in smaller size immature fruits whereas the lowest content was found in mature fruits. The high-performance liquid chromatography (HPLC) chromatograms of 12 mm and 14 mm dropped fruits which retained maximum hesperidin content of 27.03% and 27.20% after freeze drying and with recovery percentage of 17.99% after the hot air drying technique are given in Figure 2. This shows that the compounds are not detected viz. Narirutin/isonaringin, diosmin, didymin/neoponcirin in samples dried by the hot air oven technique. The immature fruits were found to have the highest hesperidin content at an early stage of fruit growth [10]. The concentration of flavonoids decreases with increase in fruit size as well as maturity [21]. The highest amount of flavonoids viz. hesperidin and naringin were reported in immature fruits of orange and grapefruit [22,23]. Similar findings of hesperidin to be accumulated in very young tissues of fruits were also reported [24,25,26].

The relative percentage of flavonoid narirutin/isonaringinanalysed ranged from 0.08% to 0.38% in different sizes of freeze dried dropped immature fruits. The same observation is made in case of diosmin and didymin/neoponcirin content and varied from 0.29% to 1.06% and 0.34% to 0.77%, respectively which decreases with increasing fruit diameter (Table 1). A similar observation was reported in immature fruits as compared to mature ones [27]. In citrus fruits, the content of flavonoids per fruit weight decreases with fruit growth and was found maximum in young developing fruits [28,29]. Similar results were obtained while quantifying the flavonoid content in grapefruit and pummelo [22]. Interestingly it was observed that the flavonoids namely narirutin/isonaringin, diosmin and didymin/neoponcirin were not detected in hot air dried samples. From the study conducted and results obtained, correlation was found between fruit growth developmental stages and flavonoid content. The freeze drying technique can be suggested as an alternative to the hot air drying technique for maximum retention of flavonoid content in dried material.

### 2.2. Antioxidant Activity

**Figure 3:**
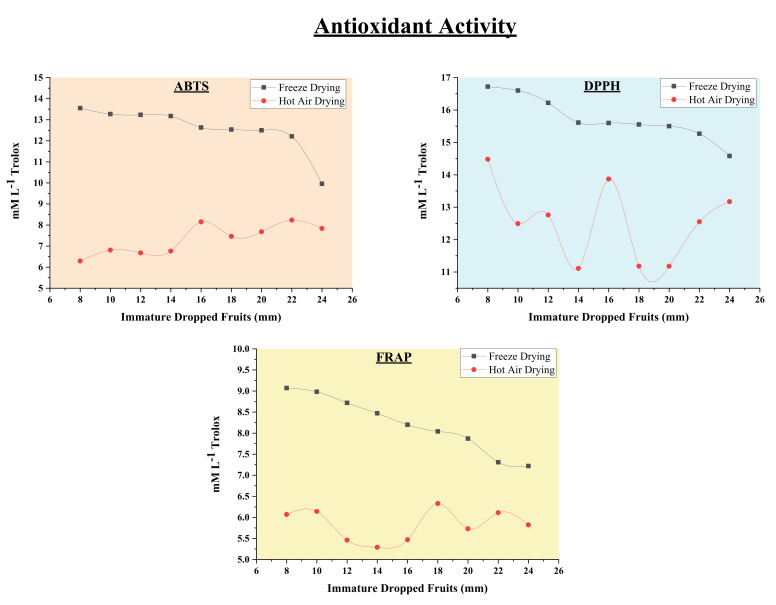
Antioxidant activity (ABTS, DPPH and FRAP assays) of immature mandarin (*Citrus reticulata* Blanco) dropped fruits after freeze drying and hot air oven drying.

The free radicals are harmful species causing oxidation of important biomolecules like proteins, DNA, etc. responsible for causing diseases. Antioxidants exert their action by scavenging and blocking free radicals and protect and preventing from various diseases like diabetes, cancer, inflammation, Alzheimer’s disease, etc. [19]. To screen the potential application of *Citrus reticulata* Blanco immature dropped fruits, we carried out determination of antioxidant activity. The antioxidant assays of azino-bis [3-ethylbenzthiazoline-6-sulfonic acid]) (ABTS), 2,2-diphenyl-1-picrylhydrazyl radical (DPPH) and ferric reducing ability of plasma (FRAP) were adopted in the present study. In ABTS and DPPH assays, the antioxidant compound quench the ABTS·^+^ and bleach a 2,2-diphenyl-1-picrylhydrazyl solution converting to a colorless product. The degree of discoloration of ABTS and DPPH depends on the scavenging ability of sample [30]. The greater the discoloration, greater is the antioxidant activity of the sample. Antioxidant potential by ABTS and DPPH assays is depicted in Figure 3 and the results are reported in mM L^−1^ Trolox. The antioxidant activity by ABTS assay ranged from 12.21–13.554 mM L^−1^ Trolox and 6.68–8.15 mM L^−1^ Trolox and that measured by DPPH assay ranged from 15.27–16.72 mM L^−1^ Trolox and 11.11–14.48 mM L^−1^ Trolox respectively when dried using vacuum freeze dryer and hot air oven. It was observed that among the different drying techniques employed; the highest antioxidant activity is seen in fruits dried using vacuum freeze dryer as compared hot air drying. The hot air drying resulted in reducing the antioxidant potential of mandarin immature dropped fruits. The similar effect of drying while studying antioxidant activity of citrus fruits, *Leccinum scabrum* (Bull.) Gray and *Hericium erinaceous* (Bull.) Pers. [14] was also observed. The scavenging activity in immature kumquats was found to be higher than mature kumquats [31] and similar observations were also reported while determining antioxidant activity of chinotto [6] and immature *Citrus unshiu* fruits [19], respectively. The antioxidant activity of the physiologically dropped immature *Citrus reticulata* Blanco fruits evaluated by FRAP assay gave a similar trend as observed in ABTS and DPPH assays. The FRAP values ranged from 7.31–9.07 mM L^−1^ Trolox (freeze dried) and 5.29–6.33 mM L^−1^ Trolox (hot air dried). The highest antioxidant potential was observed in immature fruits (Figure 3). Researchers have reported that potential radical scavenging activities are seen in citrus fruits and peel [32,33].

### 2.3. Total Phenol Content

**Figure 4:**
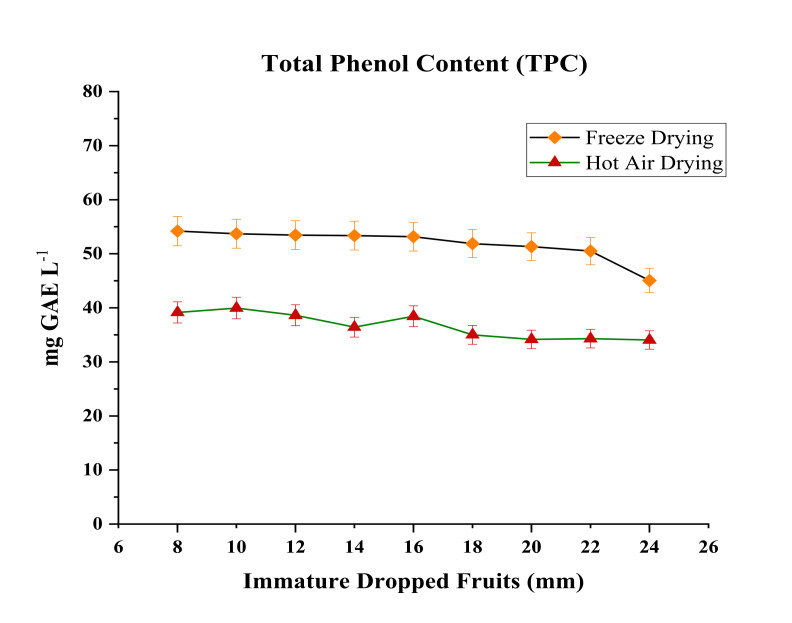
Total phenol content (TPC) of immature mandarin (*Citrus reticulata* Blanco) dropped fruits after freeze drying and hot air oven drying.

The total phenolic content (TPC) of nine different sizes of immature dropped fruits was measured (Figure 4). TPC plays an important role in determining antioxidant activity [5]. More antioxidant activity is found in fruits having higher phenolic content [34]. The TPC content in our study varied from 50.50–54.19 mg GAE L^−1^ in freeze dried dropped fruit and 34.04–39.95 mg GAE L^−1^ in hot air dried dropped fruits respectively. The antioxidant potential was found higher in immature dropped fruits (8–16 mm). In a study, it was reported that the concentration of polyphenolics in the fruit is influenced by the maturity [35]. Several reports showed greater antioxidant activity in immature citrus fruits with respect to mature fruits [6,36]. A high amount of phenolic content were found in fruits dried in vacuum freeze dryer and a reduction was observed after the drying process in a hot air oven at 45–50 °C. Phenols are temperature sensitive molecule [14] and oxidation reactions are responsible for loss in content during drying process. In the hot air drying technique, both enzymatic and non-enzymatic oxidative reactions occur. In case of freeze-drying technique, the enzymes responsible for oxidative reactions are polyphenols oxidase (PPO) and peroxidise (POD) [37]. The lower oxygen atmosphere oxidizes the enzymes, disrupts cell structures due to formation of ice crystals and exposes the phenolic compounds to the oxidized conditions thereby changing the structure of the molecules [14,18]. Besides this, the binding of phenolic compounds to protein molecules and inability of the applied method for extraction of phenols are the reason for the loss in content [13]. Earlier analyses in grape skin [38], in citrus fruits [5] and in tomatoes and ginger [13] also reported that freeze drying results in higher phenolic compounds content than hot air drying.

From the experiment study carried out for determining the flavonoids, antioxidant activity by ABTS, DPPH, FRAP and total phenolic content (TPC), freeze drying technique can be recommended as a beneficial method for preserving the content with respect to the hot air drying method for better retention.

### 2.4. Correlation Coefficients between Flavonoid Content, Antioxidant Activity and Total Phenol Content

Based on the experimental findings of flavonoids, antioxidant activity by ABTS, DPPH, FRAP and total phenolic content, the Pearson’s correlation coefficients after the freeze drying technique was assessed and is depicted in Table 2. The antioxidant activities from hesperidin flavonoid were correlated well with the ABTS, DPPH and FRAP with correlation coefficient values (*r*) of 0.802, 0.840 and 0.959 at *p* < 0.01. Hesperidin is considered as one of the major flavanone glycoside found in the genus citrus [10]. The correlation was also seen between another flavonoid viz. diosmin with ABTS and FRAP. The correlation coefficient was 0.806 at *p* < 0.01 and 0.726 at *p* < 0.05, respectively. The positive correlation between hesperidin and diosmin were also observed (*r* = 0.767 at *p* < 0.05). Flavonoid is responsible for determining the antioxidant activity of fruit [39]. The positive correlation between TPC and hesperidin was observed (*r* = 0.821 at *p* < 0.01). A correlation coefficient of *r* = 0.854 at *p* < 0.01 was recorded between TPC and diosmin. Higher antioxidant activity is seen in fruits with higher TPC content [34]. Significant correlation both at *p* < 0.01 and *p* < 0.05 between antioxidant assays viz. include ABTS, DPPH, FRAP and total phenol content (TPC) was also seen (See Table 2). Similar findings were also obtained while studying the antioxidant activity of physiological dropped citrus cultivars grown in China [39] and citrus peel extract [40] respectively.

## 3. Materials and Methods

### 3.1. Materials

**Figure 5:**
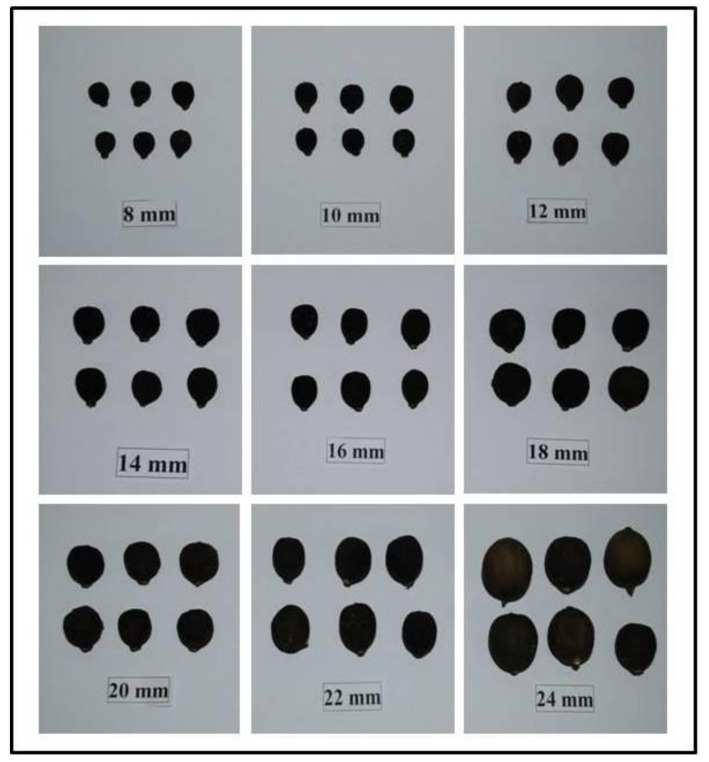
Physiologically dropped immature mandarin (*Citrus reticulata* Blanco) fruits of varying sizes from 8–24 mm.

Dropped immature mandarin (*Citrus reticulata* Blanco) fruits were collected from experimental blocks of ICAR-Central Citrus Research Institute, Nagpur (Maharashtra). Fruits of different diameter i.e., from 8 mm to 24 mm were taken during the study and collected during varying developmental stages (Figure 5).

### 3.2. Drying Techniques

The dropped fruits were washed thoroughly with distilled water to remove any dust particle adhere to it. After washing, fruits were then divided equally into two different batches for hot air oven drying and freezing drying.

### 3.3. Hot Air Oven Drying and Freeze Drying

The fruits were chopped into thin slices of 0.5 cm thick and kept in trays. During hot air oven drying, the chopped fruits of 0.5 cm thick were transferred into petri dishes and kept in microwave-oven (RIVOTEK, Reviera Glass Pvt. Ltd., Mumbai, India) with temperature of 45–50 °C for 24 h to 36 h until complete drying was achieved. The freeze drying technique was undertaken with the chopped fruits being kept in −20 °C deep freezer (NEW BRUNSWICKTM, Eppendorf, India) for 12 h and then lyophilized in vacuum freeze dryer (Mini Lyotrap, LTE Scientific Ltd., Mumbai, India) for 24 h to 48 h at −50 °C to −55 °C.

**Figure 6:**
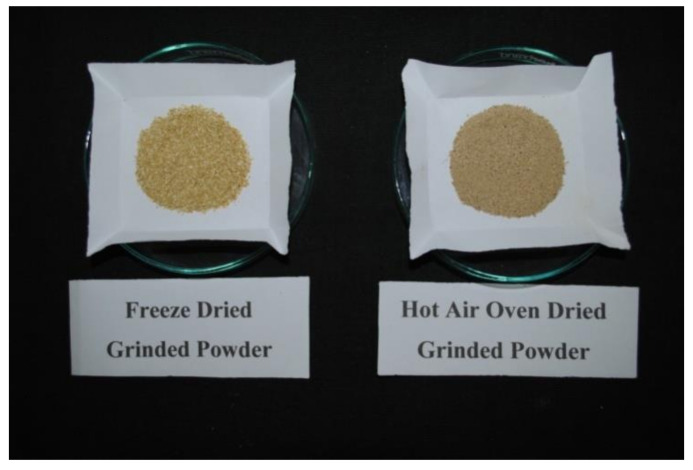
Freeze dried and hot air oven dried grinded powder of physiologically dropped immature mandarin (*Citrus reticulata* Blanco) fruits.

After different drying treatments, samples were crushed and grinded to fine powder using mortar and pestle (Figure 6). This grinded powder was then passed through a 50 micron sieve, packed in polythene bags and stored at −20 °C in a deep freezer (NEW BRUNSWICKTM, Eppendorf, India) and used for further analysis.

### 3.4. Reagents and Standards

The flavonoid standard of hesperidin, narirutin/isonaringin, diosmin and didymin/neoponcirin, antioxidant standard trolox, ABTS·^+^ (radical cation azino-biso [3-ethylbenzthiazoline-6-sulfonic acid]),o 2, o2-o diphenyl-1-picrylhydrazyl radical (DPPH), 2, 4, 6-Tri (2-pyridyl)-s-triazine (TPTZ), and gallic acid were purchased from Sigma–Aldrich (Mumbai, India). The chemicals namely ammonium acetate, acetonitrile, dimethyl sulfoxide used was of HPLC grade. Other analytical grade chemicals were used in the present study.

### 3.5. Sample and Standard Preparation

Whole fruit powder of 3 mg was taken while preparing samples for extraction and quantification of flavonoid content. Dimethyl sulfoxide (DMSO) 5 mL was added and the whole solution was sonicated for about 20 min in sonicator 2K1008008 series (Life-Care Equipments Pvt. Ltd., Mumbai, India). Then this was filtered through 0.45 micron nylon filter and injected into HPLC Agilent Model No. 1260 Infinity System (M/s. Agilent Technologies Pvt. Ltd., Bangaluru, India) for flavonoids analysis [10] and their relative percentage was reported.

Standard stock solution of 600 ppm was prepared by dissolving each flavonoid (hesperidin, narirutin/isonaringin, diosmin and didymin/neoponcirin) in dimethyl sulfoxide. For calibration purposes, stock solution was diluted to further lower concentrations using mobile phase.

### 3.6. Flavonoid Quantification by High-Performance Liquid Chromatography (HPLC)

Chromatographic separation of flavonoid in small immature mandarin (*Citrus reticulata* Blanco) fruit powder was performed using a reverse phase column, Nucleosil 100-C18, 4.6 mm, 100 mm length. The mobile phase was prepared by taking ammonium acetate (5 mM) and acetonitrile in 75:25 (*v*/*v*) ratio. pH adjusted with acetic acid. The column temperature was maintained at 40 °C. The detection wavelength was set at 284 nm and injection volume for sample as well as standard was 5 µL [41].

### 3.7. Antioxidant Activity

The antioxidant activity was assessed with 96 well plates in an automated microplate reader Tecan Infinite M200 Pro (Tecan Group Ltd., Switzerland).

#### 3.7.1. ABTS·^+^Radical Scavenging Assay

The ABTS·^+^decoloration assay was performed using the method described previously [42]. The antioxidant compound present quenches the ABTS·^+^ relative to trolox antioxidant standard used. Results were reported in mmol L^−1^ Trolox. Samples were assessed in three replicated trials.

#### 3.7.2. DPPH Radical Scavenging Assay

The radical-scavenging activity of juices was carried out [42]. The DPPH was used as stable radical. The electron donation ability was measured by bleaching of the purple colored solution of 2,2-diphenyl-1-picrylhydrazyl radical (DPPH). Results were reported in mmol L^−1^ Trolox. Samples were assessed in three replicated trials.

#### 3.7.3. FRAP Assay

The ferric reducing ability of plasma (FRAP) assay as a measure of antioxidant power was determined according to the procedure in the literature with some additional modifications [43]. In the FRAP assay, Fe (III)- tripyridyltriazine complex was reduced to the blue ferrous form by the antioxidant compounds present in the sample, which has an absorption maxima at 593 nm. The FRAP reagent was prepared with acetate buffer of 300 mM, TPTZ solution, and ferric chloride solutions in the ratio 10:1:1 and was added to a well containing 2 µL sample extract. The absorbance at 593 nm was measured at zero time and after reaction completion of 40 min, respectively. The difference in absorbance between two is proportional to the antioxidant power of the sample extract. Quantification was undertaken with respect to the standard calibration curve of trolox. Results were reported in mmol L^−1^ Trolox. Samples were assessed in three replicated trials.

### 3.8. Total Phenol Content (TPC)

The Folin–Ciocalteu method was referred for the estimation of total phenols content (TPC) [44]. Samples of 10 µL prepared for assaying total phenols was mixed with 790 µL milli-Q water and 50 µL Folin–Ciocalteu reagent and vortexed. After the addition of 150 µL sodium carbonate solution (20%), the content was quantified at 750 nm after 1 h of incubation at room temp (23.5 °C). Total polyphenol was expressed as gallic acid equivalents (mg GAE L^−1^).

### 3.9. Statistical Analysis

The results are means of three measurements. The data is presented as means ± standard deviation. Analysis of variance (ANOVA) along with Tukey’s honestly significant difference (HSD) test, which is a multiple range test, was used to determine the significance of test. To correlate relationships between parameters, Pearson correlation analysis was performed [45]. The probability values (*p*) of <0.01 and <0.05 were adopted as statistically significant.

## 4. Conclusions

The bioactive compounds and antioxidant potential of immature dropped fruits of *C. reticulata* Blanco influenced by freeze and hot air drying techniques were investigated in the present study. As flavonoids, antioxidants and total phenols have promising health benefits which now are universally accepted. Among flavanone glycosides, hesperidin was found in abundance in freeze dried samples. Among varying dropped fruits assessed, freeze dried samples of immature dropped citrus fruits, especially at the sizes of 12 mm and 14 mm, retained the composition of functional compounds in comparison with hot air dried samples. The increase in temperature significantly affects the contents of the bioactive compounds analyzed and antioxidant property assessed. The flavonoid composition especially diosmin and hesperidin correlated well at *p* < 0.01 and *p* < 0.05, respectively, with the antioxidant assays ABTS, DPPH, FRAP and with phenolic compounds. We believe that this study is important as immature fruits as waste can prove useful in preventing diseases and damage caused due to oxidative stress as they exhibit potent antioxidant activity of flavonoid compounds and phenols. The results presented in the study provide useful information on freeze-dried immature dropped citrus fruits of 12 mm and 14 mm size as a good source of potential dietary supplements with high nutraceuticals, which brings novelty to the field. The freeze-drying technique can be considered as alternative and emerging technology which can be adopted as a good option to retain the functional properties of citrus fruits.

## Figures and Tables

**Table 1:** Flavonoids content (relative percentage) in Mandarin (*Citrus reticulata* Blanco) immature dropped fruits after freeze drying (up to 48 h) and hot air drying (up to 36 h).

Sr. No.	Fruit Size (mm)	Hesperidin (%)		Narirutin/Isonaringin (%)		Diosmin (%)		Didymin/Neoponcirin (%)	
		Freeze Dried	Hot Air Dried	Freeze Dried	Hot Air Dried	Freeze Dried	Hot Air Dried	Freeze Dried	Hot Air Dried
1	8	28.48 ^a^ ± 0.16	17.40 ^a^ ± 0.05	0.22 ^c^ ± 0.01	nd	1.06 ^a^ ± 0.10	nd	0.53 ^cd^ ± 0.06	nd
2	10	26.22 ^c^ ± 0.32	10.94 ^b^ ± 0.10	0.10 ^d^ ± 0.04	nd	0.58 ^bc^ ± 0.01	nd	0.59 ^bc^ ± 0.02	nd
3	12	27.03 ^bc^ ± 0.13	17.99 ^a^ ± 0.92	0.21 ^c^ ± 0.02	nd	0.54 ^c^ ± 0.06	nd	0.65 ^b^ ± 0.01	nd
4	14	27.20 ^b^ ± 0.46	17.99 ^a^ ± 0.09	0.23 ^c^ ± 0.02	nd	0.70 ^b^ ± 0.03	nd	0.77 ^a^ ± 0.02	nd
5	16	20.93 ^d^ ± 0.11	11.34 ^b^ ± 0.05	0.38 ^a^ ± 0.02	nd	0.55 ^c^ ± 0.02	nd	0.77 ^a^ ± 0.02	nd
6	18	15.11 ^e^ ± 0.20	8.94 ^c^ ± 0.01	0.22 ^c^ ± 0.03	nd	0.30 ^d^ ± 0.01	nd	0.54 ^cd^ ± 0.04	nd
7	20	12.44 ^f^ ± 0.31	6.87 ^d^ ± 0.03	0.08 ^d^ ± 0.01	nd	nd	nd	0.35 ^e^ ± 0.02	nd
8	22	8.08 ^g^ ± 0.20	5.27 ^e^ ± 0.04	0.26 ^bc^ ± 0.01	nd	nd	nd	0.43 ^de^ ± 0.01	nd
9	24	7.76 ^g^ ± 0.12	6.57 ^d^ ± 0.03	0.33 ^ab^ ± 0.04	nd	nd	nd	0.39 ^e^ ± 0.04	nd
Tukey’s HSD at 1%		0.8794	1.0936	0.0853	-	0.1462	-	0.1084	-

nd: not detected. Data presented are in means ± standard deviation (*n* = 3). Statistical note: Means (*n* = 3) within a column followed by different letters are significantly different at *p* < 0.01 according to the Tukey’shonestly significant difference (HSD) multiple range test. * Means with superscripts having the same letter are not significantly different.

**Table 2:** Correlation coefficients between flavonoids content, antioxidant activity and total phenol content (*n* = 9) after freeze drying treatment.

	Hesperidin	Diosmin	ABTS	DPPH	FRAP	TPC
Hesperidin	-					
Diosmin	0.767 *	-				
ABTS	0.802 **	0.806 **	-			
DPPH	0.840 **	0.620	0.868 **	-		
FRAP	0.959 **	0.726 *	0.837 **	0.943 **	-	
TPC	0.821 **	0.854 **	0.987 **	0.850 **	0.843 **	-

Note: The correlation value *r* is given. Values significant at *p* < 0.01 are denoted by two asterisks and that significant at *p* < 0.05 are denoted by one asterisk. No asterisk means the correlation was not found to be significant.

## Data Availability

Samples of the data are provided by the corresponding author on request.

## Sample Availability

Samples of the compounds are available from the authors.

## References

[1] Turner T., Burri B.J. (2013). Potential Nutritional Benefits of Current Citrus Consumption. Agriculture.

[2] NHB Horticulture Database. 2018–2019. www.nhb.gov.in.

[3] Lv X., Zhao S., Ning Z., Zeng H., Shu Y., Tao O., Xiao C., Lu C., Liu Y. (2015). Citrus fruits as a treasure trove of active natural metabolites that potentially provide benefits for human health. Chem. Cent. J..

[4] Okwu D.E. (2008). Citrus fruits: A rich source of phytochemicals and their roles in human health. Int. J. Chem. Sci..

[5] Sun Y., Shen Y., Liu D., Ye X. (2015). Effects of drying methods on phytochemical compounds and antioxidant activity of physiologically dropped un-matured citrus fruits. LWT Food Sci. Technol..

[6] Barreca D., Bellocco E., Caristi C., Leuzzi U., Gattuso G. (2010). Flavonoid Composition and Antioxidant Activity of Juices from Chinotto (*Citrus* × *myrtifolia* Raf.) Fruits at Different Ripening Stages. J. Agric. Food Chem..

[7] Inafuku-Teramoto S., Suwa R., Fukuzawa Y., Kawamitsu Y. (2011). Polymethoxyflavones, Synephrine and Volatile Constitution of Peels of Citrus Fruit Grown in Okinawa. J. Jpn. Soc. Hortic. Sci..

[8] Tripoli E., La Guardia M., Giammanco S., Di Majo D., Giammanco M. (2007). Citrus flavonoids: Molecular structure, biological activity and nutritional properties: A review. Food Chem..

[9] Leo F.D., Bosco S.F.D. Citrus flavonoids as bioactive compounds: Role, bioavailability, socio-economic impact and biotechnological approach for their modification. Proceedings of the 9th ICABR International Conference on Agricultural Biotechnology: Ten Years Later.

[10] Omidbaigi R., Nasiri M.F. (2004). Quantitive distribution of hesperidin in Citrus species, during fruit maturation and optimal harvest time. Nat. Prod. Radiance.

[11] Rafiq S., Kaul R., Sofi S.A., Bashir N., Nazir F., Nayik G.A. (2018). Citrus peel as a source of functional ingredient: A review. J. Saudi Soc. Agric. Sci..

[12] Kumar D., Ladaniya M.S., Gurjar M. (2019). Underutilized Citrus sp. Pomelo (*Citrus grandis*) and Kachai lemon (*Citrus jambhiri*) exhale in phytochemicals and antioxidant potential. J. Food Sci. Technol..

[13] Gümüşay Ö.A., Borazan A.A., Ercal N., Demirkol O. (2015). Drying effects on the antioxidant properties of tomatoes and ginger. Food Chem..

[14] Gąsecka M., Siwulski M., Magdziak Z., Budzyńska S., Stuper-Szablewska K., Niedzielski P., Mleczek M. (2020). The effect of drying temperature on bioactive compounds and antioxidant activity of *Leccinum scabrum* (Bull.) Gray and *Hericium erinaceus* (Bull.) Pers. J. Food Sci. Technol..

[15] Kumar D., Ladaniya M.S., Gurjar M., Mendke S., Kumar S. (2020). Hesperidin a major flavonoid with high antioxidant potential and nutraceutical source in dropped fruits of sweet orange (C. *sinensis* (L.) osbeck). Int. J. Innov. Hortic..

[16] McClements D.J. (2015). Nanoparticle- and Microparticle-Based Delivery Systems: Encapsulations, Protection and Release of Active Compounds.

[17] Varzakas T., Zakynthinos G., Verpoort F. (2016). Plant Food Residues as a Source of Nutraceuticals and Functional Foods. Foods.

[18] Li R., Shang H., Wu H., Wang M., Duan M., Yang J. (2018). Thermal inactivation kinetics and effects of drying methods on the phenolic profile and antioxidant activities of chicory (*Cichorium intybus* L.) leaves. Sci. Rep..

[19] Kim J.H., Kim M.Y. (2017). Phytochemical and antioxidant characterization of thinned immature *Citrus unshiu* fruits. Int. J. Pharm. Pharm. Sci..

[20] Omidbaigi R., Nasiri M.F., Sadr Z.B. (2002). Hesperidin in citrus species, quantitative distribution during fruit maturation and optimal harvesting time. Possibilities Limit. Med. Aromat. Plant.

[21] Ladaniya M.S. (2008). Citrus Fruit: Biology, Technology and Evaluation.

[22] Ortuño A., Garcia-Puig D., Fuster M.D., Perez M.L., Sabater F., Porras I., Garcia-Lidon A., Del Rio J.A. (1995). Flavanone and Nootkatone Levels in Different Varieties of Grapefruit and Pummelo. J. Agric. Food Chem..

[23] Jourdan P.S., McIntosh C.A., Mansell R.L. (1985). Naringin levels in citrus tissues: II. Quantitative distribution of naringin in *Citrus paradise* Macf. Plant Physiol..

[24] Hasegawa S., Maeir V.P. Some aspects of citrus biochemistry and juice quality. Proceedings of the International Society of Citriculture.

[25] Del Rio J.A., Ortuno A. (1994). *Citrus paradise* Macf (Grapefruit): In vitro culture and the bioproduction of sesquiterpenes nootkation, valencene and other secondary metabolites. Biotechnology in Agriculture and Forestry.

[26] Del Rio J.A., Fuster M.D., Sabater F., Porras I., Garcia-Lidon A., Ortuno A. (1995). Effect of benzylaminopurine on the flavanones hesperidin, hesperetin 7-O-glucoside and prunin in tangelo nova fruits. J. Agric. Food Chem..

[27] Lou S.-N., Lai Y.-C., Hsu Y.-S., Ho C.-T. (2016). Phenolic content, antioxidant activity and effective compounds of kumquat extracted by different solvents. Food Chem..

[28] Ogawa K., Kawasaki A., Omura M., Yoshida T., Ikoma Y., Yano M. (2001). 3′,5′-Di-C-β-glucopyranosylphloretin, a flavonoid characteristic of the genus Fortunella. Phytochemistry.

[29] Del Río J.A., Gómez P., Baidez A.G., Arcas M.C., Botía A.J.M., Ortuño A. (2004). Changes in the Levels of Polymethoxyflavones and Flavanones as Part of the Defense Mechanism of *Citrus sinensis* (cv. Valencia Late) Fruits against *Phytophthora citrophthora*. J. Agric. Food Chem..

[30] Almeida M.M.B., de Sousa P.H.M., Arriaga Â.M.C., do Prado G.M., de Carvalho Magalhães C.E., Maia G.A., de Lemos T.L.G. (2011). Bioactive compounds and antioxidant activity of fresh exotic fruits from northeastern Brazil. Food Res. Int..

[31] Lou S.-N., Ho C.-T. (2017). Phenolic compounds and biological activities of small-size citrus: Kumquat and calamondin. J. Food Drug Anal..

[32] Ma Y.-Q., Ye X.-Q., Fang Z.-X., Chen J.-C., Xu G.-H., Liu D.-H. (2008). Phenolic Compounds and Antioxidant Activity of Extracts from Ultrasonic Treatment of Satsuma Mandarin (*Citrus unshiu* Marc.) Peels. J. Agric. Food Chem..

[33] Kaur R., Arora S., Singh B. (2008). Antioxidant activity of the phenol rich fractions of leaves of *Chukrasia tabularis* A. Juss. Bioresour. Technol..

[34] Rice-Evans C.A., Miller N.J. (1996). Antioxidant activities of flavonoids as bioactive components of food. Biochem. Soc. Trans..

[35] Ye X.-Q., Chen J.-C., Liu D.-H., Jiang P., Shi J., Xue S., Wu D., Xu J.-G., Kakuda Y. (2011). Identification of bioactive composition and antioxidant activity in young mandarin fruits. Food Chem..

[36] Cano A., Medina A., Bermejo A. (2008). Bioactive compounds in different citrus varieties. Discrimination among cultivars. J. Food Compos. Anal..

[37] An K., Zhao D., Wang Z., Wu J., Xu Y., Xiao G. (2016). Comparison of different drying methods on Chinese ginger (*Zingiber officinale* Roscoe): Changes in volatiles, chemical profile, antioxidant properties, and microstructure. Food Chem..

[38] De Torres C., Díaz-Maroto M.C., Hermosín-Gutiérrez I., Pérez-Coello M.S. (2010). Effect of freeze-drying and oven-drying on volatiles and phenolics composition of grape skin. Anal. Chim. Acta.

[39] Sun Y., Qiao L., Shen Y., Jiang P., Chen J., Ye X. (2013). Phytochemical Profile and Antioxidant Activity of Physiological Drop of Citrus Fruits. J. Food Sci..

[40] Xu G.H., Chen J.C., Liu D.H., Zhang Y.H., Jang P., Ye X.Q. (2007). Minerals, Phenolic Compounds, and Antioxidant Capacity of Citrus Peel Extract by Hot Water. J. Food Sci..

[41] Marten S. (2007). Determination of Naringin and Hesperidin in Fruit Juice.

[42] Mena P., García-Viguera C., Navarro-Rico J., Moreno D.A., Bartual J., Saura D., Martí N. (2011). Phytochemical characterisation for industrial use of pomegranate (*Punica granatum* L.) cultivars grown in Spain. J. Sci. Food Agric..

[43] Benzie I.F.F., Strain J.J. (1996). The ferric reducing ability of plasma (FRAP) as a measure of “antioxidant power”: The FRAP assay. Anal. Biochem..

[44] Singleton V.L., Rossi J.A. (1965). Colorimetry of total phenolics with phosphomolybdic-phosphotungstic acid reagents. Am. J. Enol. Vitic..

[45] Gomez K.A., Gomez A.A. (1983). Statistical Procedures for Agricultural Research.

